# The Changing Distribution of Anthocyanin in *Mikania micrantha* Leaves as an Adaption to Low-Temperature Environments

**DOI:** 10.3390/plants8110456

**Published:** 2019-10-27

**Authors:** Qilei Zhang, Junjie Zhai, Guangxin Chen, Wei Lin, Changlian Peng

**Affiliations:** Guangdong Provincial Key Laboratory of Biotechnology for Plant Development, School of Life Sciences, South China Normal University, Guangzhou 510631, China; dalei45666@163.com (Q.Z.); sdzbzjj2007@163.com (J.Z.); gxchen2019@126.com (G.C.); 13527044032@163.com (W.L.)

**Keywords:** anthocyanins, antioxidation, gas exchange, *Mikania micrantha*

## Abstract

Anthocyanins, a protective substance in plant leaves, can accumulate in large quantities under low-temperature induction. In order to explore the effect of anthocyanins in *Mikania micrantha* leaves, the Rubisco, photosynthesis, pigments, and antioxidative capacity in mature leaves (ML) and young leaves (YL) of *M. micrantha* were investigated in winter. YL were red on both the adaxial and abaxial surfaces, while ML was red on the abaxial surfaces and green on the adaxial surfaces. Compared with ML, the relative expression of the genes related to anthocyanin synthesis and anthocyanin content were significantly higher in YL. Antioxidants such as flavonoids and total phenols were found in higher quantities, and the total antioxidant capacity was also significantly higher in YL. However, in ML, the Rubisco and chlorophyll content related to photosynthesis were significantly higher. The stomata of ML displayed a larger aperture than YL, and the stomatal conductance and photosynthetic rate were significantly higher in ML. The results suggested that *M. micrantha* leaves could better adapt to the winter environment through changing the distribution of anthocyanins in leaves of different maturity.

## 1. Introduction

Low-temperature environments in winter can cause stress and damage to many plants. It can reduce the metabolic rates [1], induce degradation of proteins, and depress the activity of antioxidant enzymes [2]. Low-temperature stress can reduce photosynthetic efficiency by damaging the photosynthetic apparatus and inducing oxidative stress [3]. Many strategies have been developed in higher plants to improve tolerance to low-temperature environments [4]. Under low-temperature conditions, some plants exhibit different adaptation modes through various complex metabolic pathway networks [5,6]. One such example of this is the accumulation of nonenzymatic substances (such as anthocyanin, phenylpropanoid, and terpenoids), to reduce the oxidative stress caused by low temperatures by enhancing the antioxidant capacity in plants [7,8], and previous studies have shown that anthocyanin accumulation increased significantly in leaves of *Acmena acuminatissima* during low winter temperatures [9].

Anthocyanin is a colored antioxidant that can protect plants against many stresses caused by both biotic and abiotic factors [10]. For example, in many plant species, anthocyanin accumulation increases significantly under environmental stresses associated with light, temperature, nutrition, or drought [11,12,13]. A previous study showed that the antioxidant capacity can be improved significantly by the antioxidant function of anthocyanin in plants [14]. Overexpression of chalcone synthase (CHS) gene, a major enzyme gene in anthocyanin biosynthesis, increased anthocyanin content and reduced oxidative stress caused by high light levels in *Arabidopsis* [15]. Anthocyanidin synthase (ANS) gene, another major enzyme gene in anthocyanin biosynthesis, was found to contribute to greater sensitivity to high light levels in ANS-deficient *Arabidopsis*. Furthermore, the expression of dihydroflavonol 4-reductase (DFR) gene, a major enzyme in anthocyanin biosynthesis, increased significantly under high light stress [13]. Anthocyanin, a red substance, can absorb about 530 nm wavelength, playing an important role in photoprotection. Research in woody species found that anthocyanins on the surface of young leaves (YL) could filter part of the light energy, thus reducing the absorbance of light energy and increasing the photoprotective ability [12]. However, low light intensity leads to an inability of plants to grow because of insufficient energy [16]. Under low light conditions, the accumulation of anthocyanin in plant leaves decreased significantly [17]. In order to adapt to changes in light intensity, plants have developed certain strategies to capture and use light efficiently; for example, the chlorophyll (Chl) *a/b* ratio and leaf thickness in many plant species change under different light conditions [16,18]. There was also a reduction in the accumulation of colored substances, such as anthocyanin, under low light conditions [17].

*Mikania micrantha* H. B. K., belonging to the Asteraceae family, is a plant native to Central and South America. Currently, it is a common invasive species in many countries in Southeast Asia and the Pacific region [19]. It is a species that prefers higher temperatures and high-light environments. In order to adapt to the low-temperature (below 15 °C) environment in the winter in South China (a region where *M. micrantha* had spread into), anthocyanins were accumulated in *M. micrantha* leaves [20]. However, anthocyanin can reduce the absorbance of light by plant leaves [12], and through this mechanism, the low-light environment can affect the growth of *M. micrantha* [21]. In this study, we aimed to illustrate that the different distribution of anthocyanin in mature leaves (ML) and YL of *M. micrantha* affected the adaption to low temperatures in winter.

## 2. Results

### 2.1. Morphology Characteristics of Mature Leaves and Young Leaves

**Figure 1:**
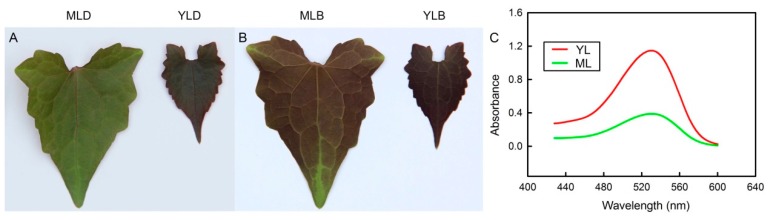
The morphology characteristics of leaves. (**A**) Adaxial surfaces of the mature leaves (MLD) and young leaves (YLD). (**B**) Abaxial surfaces of the mature leaves (MLB) and young leaves (YLB). (**C**) The absorbance of anthocyanin from mature leaves (ML) and young leaves (YL) (n = 5).

The morphology characteristics of leaves showed that the color of YL was different from that of ML. Both the adaxial and abaxial surfaces of YL were red, while the ML adaxial surfaces (MLD) were green, and the ML abaxial surfaces (MLB) were red (Figure 1A,B). The absorbance of YL was significantly higher than that of ML (Figure 1C) at 530 nm, suggesting that the anthocyanin content in YL was higher.

### 2.2. Antioxidants and Related Gene Expression of Mature Leaves and Young Leaves

**Figure 2:**
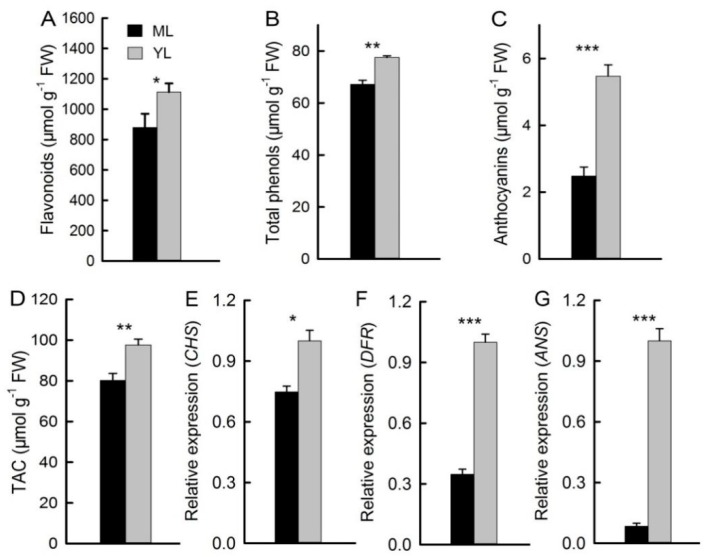
Antioxidant content of the leaves. Contents of flavonoids (**A**), total phenols (**B**), and anthocyanins (**C**) of ML and YL (n = 5). Total antioxidant capacity (TAC) of ML and YL (**G**) (n = 5). Relative expression of genes related to anthocyanin biosynthesis *chalcone synthase* (*CHS*), *dihydroflavonol 4-reductase* (*DFR*), and *anthocyanidin synthase* (*ANS*) (**D**–**F**) in ML and YL (n = 6). The error bars represent standard errors for five to six biological replicates. Asterisks indicate different significant differences (* *P* < 0.05, ** *P* < 0.01, *** *P* < 0.001) according to two-sided Student’s *t*-tests.

The anthocyanin content in YL was significantly higher than in ML (Figure 2C). The contents of flavonoids were higher in YL, and total phenols were also higher in YL (Figure 2A,B). The relative expressions of genes in the pathway of anthocyanin biosynthesis (*CHS*, *DFR*, and *ANS*) were significantly higher in YL (Figure 2E–G). In addition, the results showed that the total antioxidant capacity in YL was also significantly higher than in ML (Figure 2D).

### 2.3. Contents of Chl and Rubisco in Mature Leaves and Young Leaves

**Figure 3:**
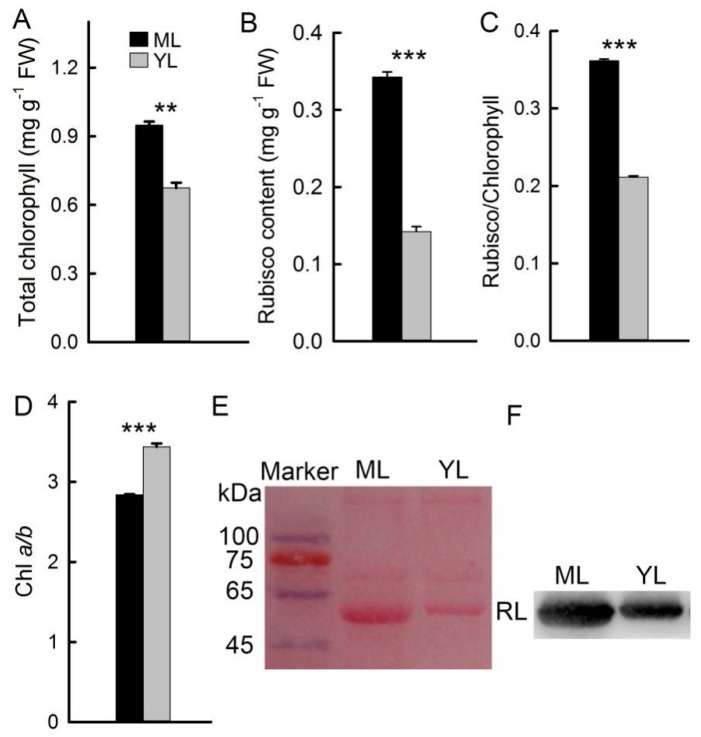
Contents of protein and chlorophyll. Contents of total chlorophyll (Chl) (**A**) and Rubisco (**B**) of ML and YL (n = 5). The values of Rubisco/chlorophyll (**C**) and Chl *a/b* (**D**) of ML and YL (n = 5). Ponceau-stained membrane before Western blot analysis (**E**) and the Western blotting analyzed Rubisco large subunit (RL) of ML and YL (**F**). The error bars represent standard errors for five biological replicates. Asterisks indicate different significant differences (** *P* < 0.01, *** *P* < 0.001) according to two-sided Student’s *t*-tests.

The contents of Chl and Rubisco related to photosynthesis in ML were evidently higher than in YL (Figure 3A,B). The values of Rubisco/Chl in YL were significantly lower than in ML (Figure 3C). By contrast, the values of Chl *a/b* in YL were significantly higher than in ML (Figure 3D). In Figure 3E, we can see Ponceau-stained membrane before Western blot analysis. The Rubisco large subunit (RL) was analyzed using Western blotting, and it showed more RL in ML than in YL (Figure 3F).

### 2.4. Stomata and Gas Exchange of Mature Leaves and Young Leaves

**Figure 4:**
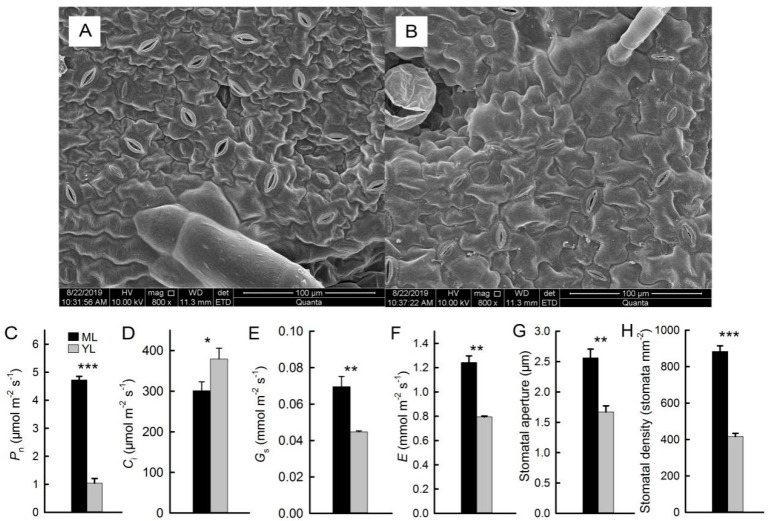
Stomata and gas exchange of leaves. Phenotypic characteristics of stomata in ML and YL (**A**,**B**). The net photosynthetic rate (*P*_n_) (**C**), intercellular carbon dioxide concentration (*C*_i_) (**D**), stomatal conductance (*G*_s_) (**E**), and transpiration rate (*E*) (**F**) of ML and YL (n = 10). Stomatal aperture and density (**G**,**H**) of ML and YL (n = 5). The error bars represent standard errors for ten biological replicates. Asterisks indicate different significant differences (* *P* < 0.05, ** *P* < 0.01, *** *P* < 0.001) according to two-sided Student’s *t*-tests.

The morphology characteristics of stomata and the parameters of gas exchange are presented in Figure 4. There were more stomata in ML compared with YL, and stomatal apertures were larger in ML (Figure 4A,B,G,H). As the main structure mediating gas exchange between leaves and the environment, stomatal aperture can directly affect gas exchange. The results showed that stomatal conductance (*G*_s_) was lower in YL and higher in ML (Figure 4E). The values of net photosynthetic rate (*P*_n_), and transpiration rate (*E*) were consistent with the *G*_s_ (Figure 4C,F). By contrast, intercellular carbon dioxide concentration (*C*_i_) was higher in YL (Figure 4D).

## 3. Discussion

*Mikania micrantha*, a plant native to Central and South America, is both photophilic and thermophilic [20,21,22]. It turned red in order to adapt to the winter environment of the area into which it is spreading (Figure 1A,B). The reddening of *M. micrantha* leaves was mainly due to the accumulation of anthocyanin (Figure 1C). Anthocyanin accumulation can be induced by a variety of environmental factors, improving plant tolerance to biotic and abiotic stresses [8]. Our previous study showed that *M. micrantha* leaves turn red in winter in low temperatures [20]. It indicated that the low temperatures could induce the accumulation of anthocyanin in plant leaves. In winter, the YL of *M. micrantha* turned red on both the adaxial and abaxial surfaces, while the ML only turned red on the abaxial surfaces, with the adaxial surfaces turning green (Figure 1A,B). This is similar to the results of woody plants, that is, from YL to ML, the red color gradually receded [12]; however, there was also a difference—the abaxial surfaces of the ML of *M. micrantha* remained red, which may be because *M. micrantha* is sensitive to low temperatures [20]. There are two main roles of anthocyanin in plant leaves. Firstly, anthocyanin, being a colored substance, can filter light energy. Studies have shown that anthocyanin attached to the surface of YL can play a role in photoprotection [23]. Secondly, anthocyanin, being an antioxidant, can effectively scavenge reactive oxygen species; thus, it has been shown that increasing the content of anthocyanin in leaves can increase the antioxidant capacity [9].

The major enzyme genes (*CHS*, *DFR,* and *ANS*) in the anthocyanin biosynthesis pathway [24] had high levels of expression in YL (Figure 2E–G). Anthocyanins accumulated in YL and decreased significantly in ML (Figure 2C). These results may be related to the photoprotective function of anthocyanins [9]. Compared with ML, the content of Rubisco in YL was significantly lower (Figure 3B,E,F). The content of Chl related to energy capture was significantly higher in ML, and the value of Chl *a/b* also decreased in ML (Figure 3A,D), which may be due to the photosynthetic system of ML being more efficient and needing to capture more light, while YL needs relatively low light energy. It is important to maintain the balance between light reaction and carbon reaction to avoid the negative effects of excessive light [25]. The value of Rubisco/Chl increased with the maturation of leaves, indicating that the accumulation of Rubisco was slower than that of Chl during the maturation of leaves, which has also been found in previous studies on woody plants [12]. Because the accumulation rate of Chl is faster than Rubisco, it may be more likely to cause excessive light energy in YL and result in photoinhibition. Therefore, increasing anthocyanins content in YL with less efficient photosynthesis can effectively reduce light absorbance and increase the photoprotective ability. This has also been confirmed in other plants [26,27].

Compared with YL, the MLD was green, but the MLB was red (Figure 1A,B). This may be related to the low-temperature resistance in winter. Low-temperature stress can reduce the photosynthetic efficiency by damaging the photosynthetic apparatus and inducing oxidative stress [3]. Anthocyanin accumulation increased significantly in different plant tissues after low-temperature stress [11,20]. Therefore, the tolerance of plant leaves to low-temperature stress can be improved by the accumulation of anthocyanin. In this study, the anthocyanin accumulated in both YL and ML of *M. micrantha* in winter were significantly higher in immature YL (Figure 1C and Figure 2C), which may be because anthocyanin was also accumulated in the adaxial surfaces of YL to reduce the absorbance of light energy. The result was consistent with the phenotypic characteristics. Flavonoids and total phenols, which are antioxidants, accumulated in the ML and YL of *M. micrantha* in winter; the contents in YL were significantly higher than in ML, and total antioxidant capacity was also higher in YL (Figure 2A,B,D). This indicates that YL need more antioxidants in winter.

Photosynthesis is the main way for higher plants to accumulate organic matter. It is also the basis for the rapid growth and high invasiveness of *M. micrantha*. The lower *P*_n_ of YL was due to the fewer opened stomata, as was the lower content of Chl and Rubisco. The *P*_n_ of ML was significantly higher than that of YL in winter (Figure 4C), which was also consistent with the results of Rubisco and Chl. Low temperature damage to the photosynthetic apparatus limits photosynthesis in plants [17,28]. In winter, in order to improve the tolerance of leaves to low temperature, anthocyanins in ML of *M. micrantha* did not disappear completely but were retained in the abaxial surfaces of leaves (Figure 1B), which meant a large number of antioxidants were accumulated in the leaves, thus improving the antioxidant capacity. The results of *G*_s_ and *E* were consistent with *P*_n_ in YL because the stomata apertures were smaller [29]. However, the *C*_i_ was significantly higher in YL due to the lower *P*_n_ of YL; however, YL had a higher respiratory rate than ML [9,12].

## 4. Materials and methods

### 4.1. Plant Materials

*M. micrantha* was planted in the biological garden of South China Normal University, Guangzhou, China. The method of planting was that of vegetative reproduction. The *M. micrantha* branches were cut into lengths of about 10 cm with two stem nodes after the leaves had been cut off; these were planted into soft soil that had been turned over, weeded, and routinely watered. The YL and ML of *M. micrantha* exhibited a striking redness in December (the mean temperature and photosynthetic photon flux density were 12.6 °C and 1100 μmol m^−2^ s^−1^, respectively). YL were red on both the adaxial and abaxial surfaces, while ML were green on the adaxial surfaces and red on the abaxial surfaces. ML and YL of *M. micrantha* were selected as research materials.

### 4.2. Pigment Estimation

Firstly, 0.1 g fresh leaves of *M. micrantha* were weighed, put into a 5 mL centrifugal tube with 4 mL 1% methanol hydrochloride (*v*/*v*), and then left in darkness at 4 °C for 24 h [30,31]. A new 4 mL centrifugal tube was selected, and a 2 mL sample with 2 mL chloroform and 1 mL ddH_2_O were added successively. After mixing, the mixture was divided into two parts and the anthocyanin was dissolved in the upper. The absorbance of the anthocyanin extract at 420–700 nm was determined by ultraviolet spectrophotometer UV-2450 (Shimadzu, Tokyo, Japan). The anthocyanin content in the leaves was calculated by the absorbance of anthocyanin extract at 530 nm using different concentrations of cyanidin-3-glucoside as a standard curve.

The quantity of total phenols was determined by the Folin–Ciocalteu method, as described in a previous study [32]. Firstly, 0.1 g fresh leaves of *M. micrantha* were weighed and put into a 2 mL centrifugal tube with 1.5 mL 95% methanol and then left in darkness at 4 °C for 24 h. A new 4 mL centrifugal tube was selected, and a 0.5 mL sample with 1 mL 10% Folin–Ciocalteu and 2 mL Na_2_CO_3_ were added successively. After mixing for 5 min, the ultraviolet spectrophotometer UV-2450 (Shimadzu, Tokyo, Japan) was used to detect the absorbance of the mixture at 765 nm. The standard curves of different concentration of Gallic acid were drawn, and the absorbance of the mixture at 765 nm was used to calculate the total phenol content in the leaves using the standard curves.

The content of flavonoids was determined using the method described by Heimler et al. (2005) [33]. Firstly, 0.1 g fresh leaves of *M. micrantha* were weighed, put into a 2 mL centrifugal tube with 1.5 mL 95% methanol, and then left in darkness at 4 °C for 24 h. A new 4 mL centrifugal tube was selected, and a 0.15 mL sample with 1.85 mL ddH_2_O, 0.2 mL 5%NaNO_2_, 0.3 mL 10% AlCl_3_, and 1 mL 1 M NaOH were added successively. After mixing, the mixture was analyzed by ultraviolet spectrophotometer UV-2450 (Shimadzu, Tokyo, Japan) at 510 nm. The standard curves of different concentrations of Catechin were drawn, and the absorbance of the mixture at 510 nm was used to calculate flavonoids contents in the leaves using the standard curves.

The content of chlorophyll was determined as follows. Firstly, 0.1 g fresh leaves of *M. micrantha* were weighed, put into a 5 mL centrifugal tube with 4 mL 80% acetone, and then left in darkness at 4 °C for 24 h. Ultraviolet spectrophotometer UV-2450 (Shimadzu, Tokyo, Japan) was used to detect the absorbance of the extract at 663 and 645 nm. The chlorophyll (Chl) content in the leaves was calculated according to Wellburn (1994) [34].

### 4.3. Total Antioxidant Capacity Determination

The total antioxidant capacity (TAC) of the leaves was determined according the method described by Saha et al. (2008) [35]. Firstly, 0.1 g fresh leaves of *M. micrantha* were weighed, put into a 2 mL centrifugal tube with 1.5 mL 95% methanol, and then left in darkness at 4 °C for 24 h. A new 4 mL centrifugal tube was selected, and a 0.1 mL sample extract and 2.9 mL 120 μM 1,1-diphenyl-2-picrylhydrazyl (DPPH) were added successively. After mixing for 10 min, an ultraviolet spectrophotometer UV-2450 (Shimadzu, Tokyo, Japan) was used to detect the absorbance of the mixture at 517 nm. The standard curve was drawn using DPPH of different concentrations, and the standard curve was used to calculate the TAC of the leaves for the absorbance at 517 nm.

### 4.4. Gene Expression Analysis

Firstly, 0.1 g fresh leaves were weighed and ground in a mortar with liquid nitrogen. The RNA in the leaves was extracted by TRIzol (Invitrogen, California, MA, USA) reagent, according to the manufacturer’s instructions. The TopScript RT DryMIX (dT18) Kit (Enzynomic, Daejeon, Korea) was used to synthesis the cDNA. The relative expression of the genes was detected using a Real-Time PCR System (CFX96, Bio-Rad, California, USA). The reaction system and the SYBR Premix Ex TaqTM II Kit (Takara, Tokyo, Japan), according to the manufacturer’s instructions, were used to analyze the relative expression of the chalcone synthase protein gene (*CHS*), the *dihydroflavonol 4-reductase* protein gene (DFR), and the *anthocyanidin synthase* protein gene (*ANS*). Relative gene expression was calculated according to the 2^−ΔΔ*C*T^ method [36]. The *Actin* gene was used as an internal reference; the primer pairs were: forward: 5′-TGAAATACCCCATTGAGCATGG-3′, and reverse: 5′-GAATCCAGTACAATACCTGTGGTAG-3′. The primer pairs for the *CHS* gene were forward: 5′-ACATGCCTGGTGCAGATTACCA-3′, and reverse: 5′-AAGTGGGAATCGGAAGGTCCAC-3′. The primer pairs for the *DFR* gene were forward: 5′-AGCTTTGATGAAGCCATTSAAGGTTGC-3′, and reverse: 5′-TTCTTCACTGTCTTGGCTTT-3′. The primer pairs for the *ANS* gene were forward: 5′-TCAGCCGGTTGAAGAGAAGGAG-3′, and reverse: 5′-GAGGGCCAAATGGTCAAATCACGT-3′.

### 4.5. Rubisco Content

Firstly, 0.05 g fresh leaves of *M. micrantha* were weighed into a mortar, 1.5 mL of protein extract (60 mM Tris-HCl (pH 7.8)) buffer was added, containing 0.1% (w/v) NaCl, 2% (*v*/*v*) glycerol, 5% (w/v) polyvinyl pyrrolidone (PVP), which was then ground on ice. After grinding, the protein extract was put into a 2 mL centrifugal tube at 4 °C centrifugation for 10 min at 12,000× *g*, and the supernatant was the protein extract. Thereafter, 100 μL protein extract was absorbed, 100 μL protein loading buffer was added, and it was then boiled for 5 min. The boiled protein mixture was added to 12% SDS-PAGE gel and separated by Mini-PROTEAN 3 system (Bio-Rad, California, USA). Then, 12% SDS-PAGE was photographed with GelDoc-It Imaging System (UVP, Upland, CA, USA). The location of Rubisco size subunits was determined using a protein marker, and the Rubisco content was calculated by Total Lab Quant software (Total Lab, Newcastle upon Tyne, UK).

The detection of the Rubisco large subunit (RL) was performed by Western blotting. The polyvinylidene fluoride (PVDF) membrane was used to blot the proteins in 12% SDS-PAGE gel. The PVDF membrane with RL was dyed with Ponceau, and the protein bands were recorded using a digital camera. After cleaning the Ponceau dye on the PVDF membrane with TBST buffer (0.5 mL of Tween20, 20 mL of 1 M Tris-HCl pH = 8.0, and 8.8 g of NaCl were added to 1 L of water), it was soaked in 5% skimmed milk powder with TBST buffer for 1.5 h at 25 °C. After cleaning the PVDF membrane with TBST buffer twice (4 min each time), the PVDF membrane was soaked in anti-Rubisco antibody (Bioss, Beijing, China), which had been diluted 1500 times, for 50 min at 25 °C. After cleaning the PVDF membrane with TBST buffer 3 times (4 min each time), the PVDF membrane was soaked in goat antirabbit horseradish peroxidase-conjugated secondary antibody (Bioss, Beijing, China), which had been 3000 times diluted, for 45 min at 25 °C. After cleaning the PVDF membrane with TBST buffer twice (4 min each time), RL on the PVDF membrane was illuminated by HRP substrate (Millipore, Billerica, USA) and recorded with Tanon 5200 (Tanon, Shanghai, China).

### 4.6. Gas Exchange

The gas exchange parameters of *M. micrantha* leaves were measured by Li-6800 Portable Photosynthesis System (LI-COR, Lincoln, Nebraska, USA). The time of measurement was from 9:00 to 11:00 in the morning on a sunny day. The temperature and humidity of the leaf measurement chamber were set at 20 °C and 45%, respectively, and the light intensity was set at 800 μmol m^−2^ s^−1^ (the ratio of red to blue light was 9:1). The gas exchange parameters of the leaves were recorded when they were relatively stable.

### 4.7. Stomatal Observations

Leaves of *M. micrantha* were immersed in a fixed solution (consisting of 2% polyformaldehyde and 2.5% glutaraldehyde) at 4 °C for 12 h. Stepwise dehydration of fixed *M. micrantha* leaves was carried out with different concentrations of alcohol (30–100%). Then, the critical-point-dried (using CO_2_) leaves were sprayed with 30 nm gold. The stomatal phenotypic characteristics were observed and recorded by scanning electron microscopy (SEM) (Q25, FEI, Oregon, USA), and stomatal aperture and density were measured with ImageJ software.

### 4.8. Statistical Analysis

SPSS Statistics 19.0 (IBM, New York, NY, USA) software was used to analyze the data. The method adopted was Student’s *t*-test. Mean results were considered to be significantly different at the level *p* < 0.05. Sigmaplot 12.5 (Systat Software Inc., Richmond, USA) software was used to map the statistical data. The data shown in the figure are means ± standard errors.

## 5. Conclusions

In summary, in order to adapt to the winter environment in the region into which it was spreading, *M. micrantha* leaves accumulated anthocyanin, a red colored substance, showing redness in their leaves because of it. The contents of anthocyanin and antioxidants were significantly higher in YL than in ML, which improved the low-temperature protection. However, the photosynthetic system of YL was less efficient due to less protection by the epidermis during the maturation of YL. Increasing anthocyanin content on the adaxial surfaces of YL can reduce light absorbance and improve photoprotection. In ML, the photosynthetic system of *M. micrantha* leaves is more efficient, and more light energy is needed to increase the accumulation of organic matter, fade the anthocyanins on the adaxial surfaces of the leaves, and improve the ability of light capture. However, low temperatures in winter can cause oxidative stress on leaves, so the abaxial surfaces of ML retained anthocyanins. This additionally increased the contents of other antioxidants in the leaves, which improved the antioxidant capacity of ML. *M. micrantha* leaves, from young to mature, can better adapt to the winter environment of the areas into which they are spreading through the change of anthocyanins at different stages of development, which is conducive to the invasive nature of *M. micrantha*.
